# Soluble TWEAK independently predicts atherosclerosis in renal transplant patients

**DOI:** 10.1186/1471-2369-14-144

**Published:** 2013-07-12

**Authors:** Kultigin Turkmen, Halil Zeki Tonbul, Fatih Mehmet Erdur, Aysun Toker, Zeynep Biyik, Huseyin Ozbiner, Abduzhappar Gaipov, Elvin Enes Gul, Mehmet Kayrak, Yalcin Solak, Orhan Ozbek, Suleyman Turk, Adrian Covic

**Affiliations:** 1 Department of Nephrology, Erzincan University Mengucek Gazi Training and Reseach Hospital, Erzincan, Turkey; 2 Department of Nephrology, Necmettin Erbakan University Meram School of Medicine, Konya, Turkey; 3 Department of Radiology, Necmettin Erbakan University Meram School of Medicine, Konya, Turkey; 4 Department of Biochemistry, Necmettin Erbakan University Meram School of Medicine, Konya, Turkey; 5 Department of Cardiology, Necmettin Erbakan University Meram School of Medicine, Konya, Turkey; 6 Nephrology Clinic, Dialysis and Renal Transplant Center, ‘C.I. PARHON’ University Hospital, ‘Gr. T. Popa’ University of Medicine and Pharmacy, Iasi, Romania

**Keywords:** sTWEAK, Neutrophil-to-lymphocyte ratio, Carotid intima-media thickness, Renal transplantation

## Abstract

**Background:**

Cardiovascular risk is increased in the early stages of chronic kidney disease (CKD) and also found to be ongoing in renal transplant (Rtx) patients. As a sign of atherosclerosis, increased carotid intima-media thickness (CIMT) has been widely accepted as a strong predictor of cardiovascular disease (CVD) and mortality in CKD patients. A novel markers, soluble tumor necrosis factor-like weak inducer of apoptosis (sTWEAK) and neutrophil-to-lymphocyte ratio (NLR) were introduced as potential markers in inflammatory disorders including CKD. The role of Rtx in terms of atherogenesis is still unclear. We aimed to investigate the relationship between sTWEAK, NLR and CIMT in Rtx patients without overt CVD and to compare these results with those obtained from healthy subjects.

**Methods:**

Cross-sectional analysis in which CIMT measurements, NLR and serum TWEAK levels were assessed in 70 Rtx patients (29 females; mean age, 40.6 ± 12.4 years) and 25 healthy subjects (13 females, mean age; 37.4±8.8 years).

**Results:**

sTWEAK levels were significantly decreased (p=0.01) and hs-CRP, NLR and CIMT levels of Rtx patients were significantly increased compared to healthy subjects (p<0.0001, p=0.001, p<0.0001, respectively). sTWEAK was also found to be decreased when eGFR was decreased (p=0.04 between all groups). CIMT was positively correlated with sTWEAK and NLR in Rtx patients (r=0.81, p<0.0001 and r=0.33, p=0.006, respectively). sTWEAK was also positively correlated with NLR (r=0.37, p=0.002). In the multivariate analysis only sTWEAK was found to be an independent variable of increased CIMT.

**Conclusion:**

sTWEAK might have a role in the pathogenesis of ongoing atherosclerosis in Rtx patients.

## Background

Despite improvements in diagnostic tools and therapeutic approaches, premature death related to cardiovascular disease (CVD) remains the most common cause of death in patients with chronic kidney disease (CKD) [1]. Cardiovascular risk is increased even in the early stages of CKD and this heightened risk is also found after renal transplantation (Rtx) [2,3]. Besides traditional risk factors including hypertension, diabetes, dyslipidemia, advanced age and left ventricular hypertrophy (LVH), novel risk factors such as endothelial dysfunction (ED), vascular calcifications, oxidative stress, and inflammation are highly prevalent and seem to play a more important role in renal patients compared to healthy subjects [4-7]. Several studies suggested that persistent systemic inflammation could be the main factor responsible for the increased risk in ESRD patients, regardless of the renal replacement therapy [8]. To prove this hypothesis, researchers analyzed a large panel of biomarkers to fully characterize the relation between inflammation and CVD, including C-reactive protein, interleukin (IL)-1β, IL-6, tumor necrosis factor-α (TNF- α) [8,9], as well as several interesting new biomarkers.

Neutrophil-to-lymphocyte ratio (NLR) is a potential marker for inflammation in cardiac and non-cardiac disorders [10-12] that was also shown to be a predictor of long term mortality in patients who underwent percutaneous coronary intervention [13]. We recently demonstrated that NLR could predict inflammation in ESRD patients [14]. Recently, a novel marker - the soluble tumor necrosis factor-like weak inducer of apoptosis (sTWEAK, TNFSF12) was introduced as a tumor necrosis factor (TNF) related cytokine in various inflammatory and non-inflammatory disorders [15]. To date, a transmembrane protein, fibroblast growth factor-inducible 14 (Fn14) and a scavenger receptor, CD163 were discovered as receptors of sTWEAK [16,17]. Binding of sTWEAK to Fn14 mediates multiple effects, including cellular growth, proliferation, migration, differentiation, apoptosis, angiogenesis, fibrogenesis and inflammation [18]. A soluble form of CD163 was also discovered and introduced as a risk factor for long term mortality in patients with peripheral artery disease [19].

Since the association between TWEAK and subclinical atherosclerosis was demonstrated in general and CKD populations [20,21], we aimed to investigate the relationship between sTWEAK, NLR and carotid IMT (CIMT) in Rtx patients without overt CVD and to compare these results with those obtained from healthy subjects.

## Methods

The study protocol was approved by the Medical Ethics Committee of Selcuk University (Meram School of Medicine, Konya, Turkey). Written informed consent was obtained from all subjects included in the study.

This was a cross-sectional study involving 70 Rtx patients (29 females, 41 males; mean age, 40.6 ± 12.4 years) followed for at least 6 months in the transplantation unit of the Necmettin Erbakan University and 25 healthy subjects (13 females; mean age, 37.4 ± 8.8 years) between January and November 2011.

Patients aged 18–70 years willing to participate in the assessment of CIMT by carotid duplex ultrasonography were screened. A review of medical records (including information on age; sex; weight; medications; primary disease of ESRD) was undertaken. Exclusion criteria were: (i) angina pectoris and/or documented coronary artery disease, (ii) congestive heart failure; (iii) active infection; (iv) autoimmune disease; (v) severe secondary hyperparathyroidism (patients with iPTH > 500 pg/mL); (vi) nephrotic-range proteinuria and vii) patients with HIV, HBV and HCV history. Eighty-six RTx patients were screened and 16 patients were excluded from the study. Of the 16 excluded patients - 7 had documented coronary artery disease, 4 congestive heart failure (New York Heart Association (NYHA) class III–IV); 2 active infection; 2 secondary hyperparathyroidism; and 1 patient had autoimmune disease. None of the patients included in the study had nephrotic-range proteinuria and arrhythmia based on electrocardiography (ECG).

Twenty-five age and sex-matched normotensive healthy individuals (13 females, mean age; 37.4 ± 8.8 years) referred from outpatient clinics of the Internal Medicine Department of Necmettin Erbakan University were enrolled as control subjects. They were subjected to the same inclusion and exclusion criteria as the transplanted patients.

Blood pressure was measured in the upright sitting position after ≥5 min of rest using an Erka sphygmomanometer (PMS Instruments Limited, Berkshire, UK) with an appropriate cuff size. Two readings were recorded for each individual. The mean value of the two readings was defined as the blood pressure. Patients with SBP and DBP ≥140 mmHg and ≥90 mmHg respectively were assumed to be hypertensive. Forty-two patients were taking anti-hypertensive drugs (all patients were on verapamil). Eleven patients were taking an oral calcium-vitamin D combination, and 9 patients were on active vitamin D. Ten patients (14.3%) were on intensive insulin and 16 (22.9%) were on oral anti-diabetic medication. All 70 Rtx patients, were on prednisolone and mycophenolate mofetil (MMF), 60 (85.7%) were on tacrolimus, 4 (5.7%) were on cyclosporine-A (Cyc-A), 2 were on sirolimus (2.9%) and 4 (5.7%) were on everolimus therapy. The donor source was living-related in 32 (45.7%), living-unrelated in 4 (5.7%) and deceased-donor in 34 (48.6%) patients. None of the patients had transplant nephrectomy.

### Biochemical analyses

Venous blood samples were drawn after an overnight fast and stored at -80°C for biochemical analyses. Serum creatinine, urea, aspartate aminotransferase (AST), alanine aminotransferase (ALT), calcium, albumin, uric acid, total cholesterol (TC), high density lipoprotein cholesterol (HDL-C), triglyceride (TG) and phosphorus (P) were determined using a Synchron LX20 system (Beckman Coulter, USA) with original Beckman reagents. HDL-C levels were determined by a direct enzymatic method, without precipitation. LDL-C levels were calculated using the Friedewald formula [22]. Serum hs-CRP levels were measured with a high sensitive immunoturbidimetric assay (Diasis Diagnostic System) using an automated clinical chemistry analyzer. Normal range reference interval of hs-CRP for adults was accepted as <10 mg/L.

### STWEAK measurements

Serum TWEAK levels were measured, in both patients and healthy controls, by a commercially available, kit based on enzyme-linked immunosorbent assay (eBiosience, Human TWEAK Instant Elisa, Cat no: BMS2006INST). The results were expressed as pg/mL. The calculated overall intra-assay coefficient of variation (CV) was 7.9%.

### Definition of NLR

Complete blood counts with automated differential counts, which included total white blood cells, neutrophils and lymphocytes, obtained at the time of admission. NLR was calculated as the ratio between neutrophils and lymphocytes, obtained from the same automated blood sample.

### Glomerular filtration rate (GFR) assesment

GFR was calculated according to the simplified version of the Modification of Diet in Renal Disease (MDRD) Study prediction equation formula, GFR=186 * Creatinine^-1.154^ * Age^-0.203^ * 1.212 (if African-American)* 0.742 (if female), as defined by Levey [23].

### Carotid intima-media thickness measurements

The carotid intima media thickness recordings were performed by a single investigator who was blinded for patient’ charateristics. The carotid arteries were evaluated with the Vivid 7 echocardiography device (General Electrics, Horten, Norway) by using a 10-MHz linear probe. The acquired images were recorded for playback analysis and were later measured off-line. The common carotid artery, the carotid bulb, and internal and external carotid arteries were visualized on both sides. The IMT of the carotid arteries were measured in the distal common carotid artery at a level 15 to 20 mm proximal to the carotid bulb. The two bright echogenic lines in the arterial wall were identified as the intima and the media. Three measurements were made for each side of the body; separate means were calculated and recorded as the right and left IMT. The intraobserver coefficient of variation for carotid IMT was 2.0%.

Abnormal CIMT was defined as CIMT ≥0.82 mm to characterize atherosclerosis [24].

### Statistical analyses

Statistical analyses were carried out using the Statistical Package for Social Sciences for Windows version 15.0 (SPSS, Chicago, IL, USA). Data are expressed as the mean ± SD, with a significance level of P < 0.05. The normal distribution of all variables was tested using the Kolmogorov-Smirnov Test. Dichotomous variables were compared using the chi-square test. Statistical differences between parametric data of two groups were analyzed using the Student’s *t*-test. The Mann–Whitney U test was used to determine differences between non-parametric data. The non-parametric Spearman coefficient of correlation was used to assess correlations between variables without normal distribution. The Rtx patients were divided into tertiles of eGFR. Differences among tertiles were analyzed by one-way ANOVA.

Multivariate linear regression analyses were undertaken to determine independent associations among CIMT and other variables. Age, serum phosphorus, LDL-cholesterol, hs-CRP, NLR, and sTWEAK were entered into the regression model as independent variables, and CIMT was entered as a dependent variable. The backward elimination method was preferred in the stepwise regression analysis and p>0.1 used as a criterion for elimination in this model. At the end of the fifth step, only sTWEAK was remained statistically significant in the model. p< 0.05 was considered significant for all tests.

## Results

### Patients’ baseline characteristics

The baseline characteristics of the 70 Rtx patients and 25 healthy subjects are depicted in Table 1. The etiology of renal disease in Rtx patients was chronic glomerulonephritis (n=6, 8.6%), diabetic nephropathy (n=6, 8.6%), hypertensive nephropathy (n=13, 18.6%), polycystic kidney disease (n=2, 3.3%), tubulointerstitial nephritis (n=4, 5.7%), amyloidosis (1, 1.4%) and unknown etiology (n=38, 53.8%). Of 70 Rtx patients, 42 (60%) had post-transplant hypertension, 47 (67.1%) had post-transplant dyslipidemia, and 20 (28.6%) had new-onset diabetes after transplantation (NODAT). There were no differences in age, gender, biochemical parameters including serum LDL and HDL cholesterol, AST, ALT and phosphorus between Rtx patients and healthy subjects. Spot urine protein to creatinine ratio (PCR) values of healthy controls and Rtx patients were 0.01±0.1 and 0.3±0.2, respectively (p<0.0001). Serum glucose, urea, creatinine, uric acid, triglyceride and calcium were significantly higher in Rtx patients compared to controls (Table 1). Estimated GFR measurements were also found to be significantly lower in Rtx patients than healthy subjects (61.7±22.2 vs 119.2±32.9, respectively, p<0.0001).

**Table 1:** The baseline characteristics, clinic and laboratory features of the healthy subjects and renal transplant patients

**Parameters**	**Healthy subjects (n=25) Median (IQR)**	**Transplant patients (n=70) Median (IQR)**	**P value**
**Age (years)**	**34 (29–43)**	**38 (31–47)**	**0.17**
**Female**	**13**	**29**	**0.48**
**BMI (kg/m**^**2**^**)**	**27 (24–29)**	**24.8 (21.4-28.4)**	**0.02**
**SBP (mmHg)**	**115 (110–120)**	**122 (110–137)**	**<0.0001**
**DBP (mmHg)**	**75 (60–80)**	**80 (70–90)**	**0.001**
**Glucose (mg/dL)**	**92 (86–105)**	**92 (86–104)**	**0.04**
**Urea(mg/dL)**	**25 (19–34)**	**32 (24–39)**	**0.002**
**Creatinine (mg/dL)**	**0.7 (0.6-0.8)**	**1.1 (0.8-1.4)**	**<0.0001**
**Albumin (g/dL)**	**4.4 (4.3-4.5)**	**4.3 (4.1-4.5)**	**0.02**
**Uric acid (mg/dL)**	**3.7 (3.5-4.6)**	**5.5 (3.9-7.0)**	**<0.0001**
**AST(IU/L)**	**21 (17–22)**	**21 (17–26)**	**0.25**
**ALT(IU/L)**	**18 (15–20)**	**19 (15–25)**	**0.18**
**LDL Cholesterol (mg/dL)**	**125 (105–137)**	**118 (92–134)**	**0.38**
**HDL Cholesterol (mg/dL)**	**46 (38–50)**	**46 (38–53)**	**0.69**
**Triglyceride (mg/dL)**	**64 (88–147)**	**144 (101–181)**	**0.001**
**Calcium (mg/dL)**	**8.5 (8.5-9.4)**	**9.5 (9.1-9.9)**	**0.0001**
**Phosphorus (mg/dL)**	**3.0 (2.7-3.3)**	**3.0 (2.7-3.6)**	**0.68**
**eGFR (ml/min)**	**107 (91–144)**	**71 (53–90)**	**<0.0001**
**Spot urine**	**0.08 (0.01-0.2)**	**0.2 (0.05-0.34)**	**<0.0001**
**protein/creatinine**			
**ratio (g/mg)**			
**Hemoglobin (mg/dL)**	**13.5 (13.3-14.3)**	**14 (12.6-14.7)**	**0.70**
**Neutrophil (mm**^**3**^**)**	**3100 (2520–3515)**	**4260 (3287–5937)**	**<0.0001**
**Lymphocyte (mm**^**3**^**)**	**2190 (1690–2583)**	**2100 (1582–2710)**	**0.29**

BMI body mass index, SBP systolic blood pressure, DBP diastolic blood pressure, ALT alanine aminotransferase, AST aspartate aminotransferase, LDL low-density lipoprotein, HDL high-density lipoprotein, eGFR estimated glomerular filtration rate.

### STWEAK, NLR, hs-CRP and carotid intima media thickness measurements

Results of inflammation and atherosclerosis markers including sTWEAK, hs-CRP, NLR and CIMT are shown in Table 2 with univariate correlates of CIMT and sTWEAK in Rtx patients. sTWEAK levels were significantly decreased (p=0.01) and hs-CRP levels were significantly increased (p<0.0001) in Rtx patients compared to healthy subjects. Neutrophil count and NLR were also significantly higher in Rtx patients compared to healthy controls (p**<**0.0001, p=0.001, respectively). The mean CIMT of Rtx patients and healthy subjects were 0.72±0.28mm and 0.41±0.07mm, respectively (p<0.0001).

**Table 2:** sTWEAK, hs-CRP, NLR and CIMT values of the healthy subjects and renal transplant patients

**Parameters**	**Healthy subjects Median Median (IQR) (n=25)**	**Renal transplant patients (n=70) Median (IQR)**	**P**	**ρ (Ρ)**^**a**^	**ρ (Ρ)**^**b**^
hs-CRP (mg/L)*	**3,3 (2.25-5.8)**	**8.9 (4.97-14)**	**<0.0001**	**0,15 (p=0.25)**	**0.09 (p=0.46)**
sTWEAK (pg/mL)*	**457 (320.5-537.8)**	**388.9 (324.5-426.9)**	**0.01**	**-**	**0.81 (p<0.0001)**
**NLR**	**1.16 (0.97-1.5)**	**2.18 (1.4-2.8)**	**0.001**	**0.37 (p=0.002)**	**0.33 (p=0.006)**
**CIMT (mm)**	**0.4 (0.4-0.5)**	**0.5 (0.4-0.9)**	**<0.0001**	**0.81 (p<0.0001)**	**-**

sTWEAK soluble TNF-like weak inducer of apoptosis, NLR neutrophil-to-lymphocyte ratio, CIMT carotid intima media thickness, hs-CRP high sensitive C-reactive protein, IQR interquartile range, * represents the variables in median (IQR).

^a^ Spearman rank univariate analysis for sTWEAK in renal transplant patients.

^b^ Spearman rank univariate analysis for CIMT in renal transplant patients.

In order to analyze the values of sTWEAK, NLR, hs-CRP, and CIMT according to renal function of Rtx patients, we defined a high eGFR group (group 1) with levels above the 66th percentile (upper tertile) and a low eGFR group (group 3) with levels below the 33th percentile (lower tertile) (Table 3). NLR and CIMT were highest and sTWEAK was lowest in eGFR group 3 (Table 3). Furthermore, sTWEAK are significantly decreasing with decreasing levels / tertile of eGFR (p=0.04 between all groups). There was also significant difference in terms of sTWEAK between healthy subects and eGFR group 2 and group 3 patients (p=0.02 and p=0.03, respectively).

**Table 3:** sTWEAK, NLR, CIMT results according to eGFR groups of renal transplant patients and healthy subjects

**Parameters**	**Healthy subjects (n=25) Median (IQR)**	**eGFR group 1 (n=23) Median (IQR)**	**eGFR group 2 (n=24) Median (IQR)**	**eGFR group 3 (n=23) Median (IQR)**	p*
**MDRD (mL/min)**	**107 (52.7)**	**83 (13.56)**	**63 (8.7)**	**42 (22)**	**<0.0001**
**hs-CRP (mg/L)**	**3.3 (2.25-5.8)**	**8.7 (5.6-12.6)**	**9.45 (4.92-14)**	**9.0 (3.3-18)**	**<0.0001**
**NLR**	**1.16 (0.54)**	**2.25 (1)**	**2.16 (1.5)**	**2.58 (2.5)**	**0.005**
**CIMT (mm)**	**0.4 (0.1)**	**0.7 (0.4)**	**0.6 (0.4)**	**0.7 (0.5)**	**<0.001**
**sTWEAK (pg/mL)**	**457 (320.5-537.8)**	**399.5 (329–439)**	**382.9 (308.6-421.5)**	**361.2 (323.4-426.5)**	**0.04**

sTWEAK soluble TNF-like weak inducer of apoptosis, NLR neutrophil-to-lymphocyte ratio, CIMT carotid intima-media thickness, MDRD modification of diet in renal disease, hs-CRP high sensitive C-reactive protein, IQR interquartile range, eGFR estimated glomerular filtration rate, *p value between all groups.

### Predictors of CIMT in Rtx patients

To investigate the relationship between atherosclerosis and inflammation, we performed a univariate correlation analysis. CIMT was positively correlated with sTWEAK and NLR in Rtx patients (r=0.81, p<0.0001 and r=0.33, p=0.006, respectively). Serum sTWEAK was also positively correlated with NLR (r=0.37, p=0.002).

We also checked the relation between serum uric acid and inflammatory markers. There were no relationship between serum uric acid and inflammatory markers including hs-CRP and NLR (r=0.01, p=0.89 and r=0.05, p=0.66, respectively).

In the multivariate analysis, only sTWEAK was found to be an independent variable of increased CIMT. Regression results are shown in Table 4.

**Table 4:** The stepwise regression analysis to determine the independent predictors of carotid-intima media thickness in renal transplant patients

**Parameters**	**Standardized Beta**^**‡**^	**P value**	**95% CI**
**Step 1**			
**Age (years)**	**0.17**	**0.18**	**−0.003-0.016**
**Phosphorus (mg/dL)**	**−0.03**	**0.78**	**−0.179-0.135**
**LDL (mg/dL)**	**−0.11**	**0.37**	**−0.006-0.002**
**hs-CRP (mg/L)**	**0.13**	**0.30**	**−0.007-0.023**
**NLR**	**0.03**	**0.84**	**−0.089-0.109**
**TWEAK (pg/mL)**	**0.42**	**0.001**	**0.001-0.004**
**adjusted r**^**2**^**=0.18**			
*p=0.008			
**Step 5**			
**TWEAK (pg/mL)**	**0.42**	**<0.001**	**0.001-0.004**
**adjusted r**^**2**^**=0.20**			
*p<0.0001			

*It was define to model statistic for each step. CI Confidence interval, LDL low density lipoprotein, hs-CRP high sensitive C-reactive protein, NLR neutrophil-to-lymphocyte ratio, TWEAK tumor necrosis factor-like weak inducer of apoptosis.

^‡^ Standardized Beta means standardized regression coefficients of the variables.

## Discussion

The major findings of the present study were as follows; i) sTWEAK levels were significantly decreased and CIMT, NLR and hs-CRP were significantly increased in Rtx patients compared to healthy subjects, ii) sTWEAK was positively correlated with CIMT and NLR but not with hs-CRP in Rtx patients, iii) sTWEAK was found to be an independent predictor of increased CIMT in Rtx patients.

In recent years, sTWEAK was discovered as a multi-functional cytokine that was related to the TNF super family. Description of TWEAK in kidney was reported for the first time by Justo et al. [25] in a mouse model of folate-induced acute kidney injury. In the following years, Yilmaz et al. [26] demonstrated that a decline in eGFR was accompanied by a gradual reduction in sTWEAK in CKD patients. The same group also showed that endothelial dysfunction and decreased sTWEAK were independently associated with cardiovascular outcomes in predialytic CKD patients [21]. In addition, treatment of type 1 hypertensive diabetic CKD with amlodipin and/or valsartan improves endothelial dysfunction and normalizes sTWEAK [27]. During the preparation of the present manuscript, Gungor et al. [28] described a possible role for sTWEAK in the pathogenesis of atherogenesis in the RTx population.

In our cohort, Rtx patients had lower sTWEAK levels compared to healthy subjects. However, median values of sTWEAK levels of our Rtx patient were higher compared to CKD patients in the cohorts of Yilmaz et al. [21] and Carrero et al. [29] (388.97 pg/mL vs. 245.5 pg/mL and 208 pg/mL, respectively). We also investigated the relation between sTWEAK and kidney function in Rtx patients. After the classification of Rtx patients according to their eGFR, we found that sTWEAK levels were significantly decreased when eGFR values were decreased. These findings are in accord with those obtained by Yilmaz et al. [26]. Therefore, we hypothesized that the decrease of sTWEAK among eGFR groups in Rtx patients might be associated with ongoing inflammation in this population.

**Figure 1:**
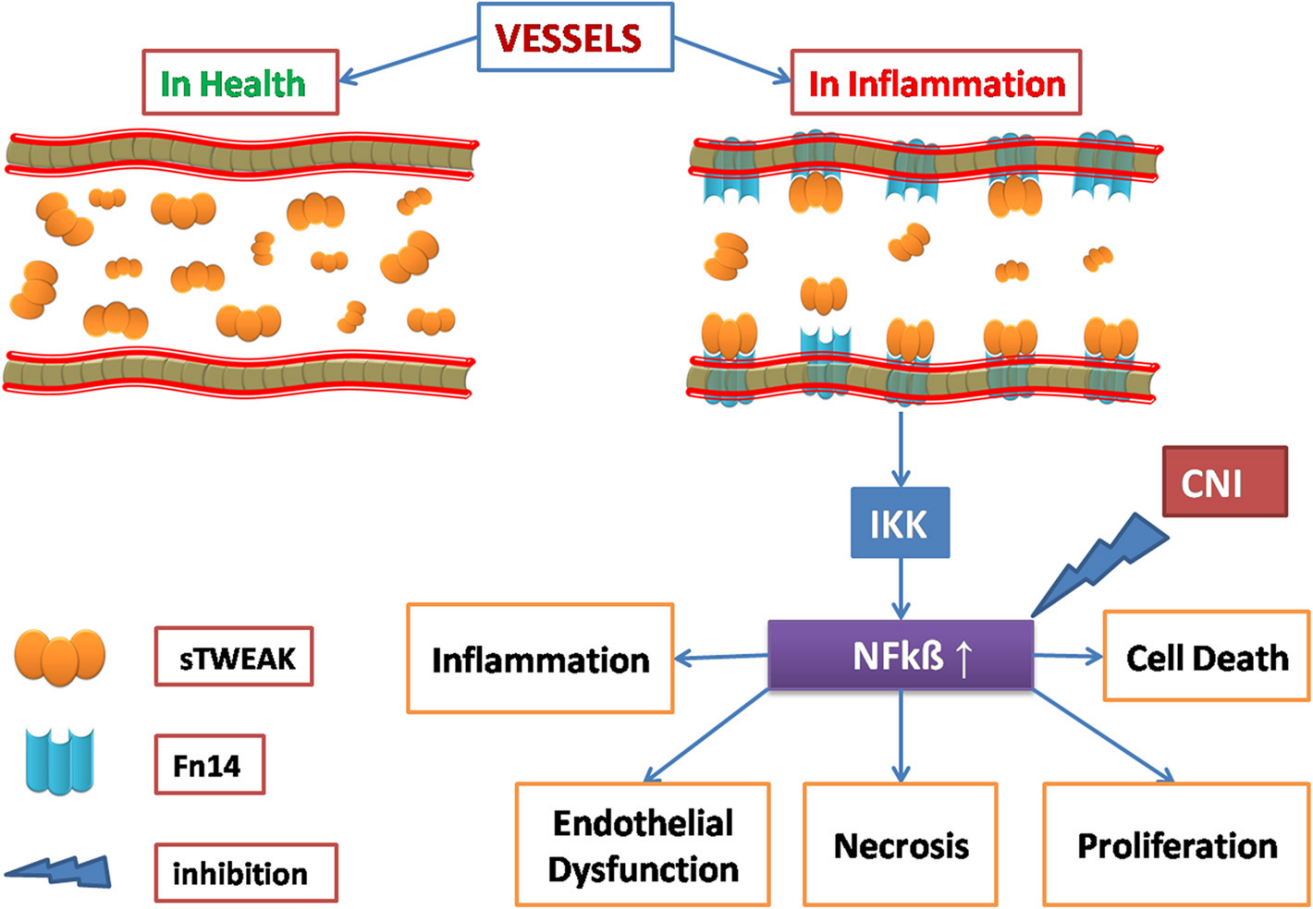
The relationship between sTWEAK and inflammation in renal transplant patients.

Inflammation seen after Rtx has a complex etiology, including a role for innate and acquired immunity. Macrophages and neutrophils play an important role in the pathogenesis of inflammation and acute and chronic allograft rejection [30]. However, ongoing inflammation in Rtx patients is much less intense compared to CKD and ESRD patients. Neutrophil-to-lymphocyte ratio was introduced as a potential marker to determine inflammation in cardiac and non-cardiac disorders [10-12]. In a previous study, we demonstrated that NLR was positively correlated with TNF- α in ESRD patients [14]. In the present study, we found that both hs-CRP and NLR were significantly increased in Rtx patients suggesting that these patients had infra-clinic, smoldering inflammation. We also showed that NLR was positively correlated with sTWEAK in this population. This finding might represent the link between inflammation and sTWEAK in Rtx patients. For better understanding, we illustrated this potential pathophysiological relationship in Figure 1.

Recently, Hornum et al. investigated the effect of Rtx on arterial function and they showed a significant improvement in endothelial function, mean arterial pressure and plasma vWF levels but not in plasma CRP and albumin levels [31]. Seyahi et al. also demonstrated that renal transplantation did not reverse or halt the progression of coronary artery calcification [32]. Our results are in accord with these studies - Rtx patients had higher CIMT compared to healthy subjects. Moreover, sTWEAK and NLR were found to be significantly and positively correlated with CIMT. Since sTWEAK is also related to eGFR, we hypothesize that decreased allograft function is associated with inflammation and atherosclerosis in a vicious circle. Therefore, sTWEAK is most probably a pathogenic cytokine rather than only a biomarker in Rtx patients.

The role of TWEAK in the renal tubulointerstitial inflammation was highlighted by Ortiz et al. [33] as an activator of nuclear factor kappa β (NFkβ), which has effects on transcription of inflammatory cytokines including MCP-1, RANTES, and IL-6 [33]. Tacrolimus, widely used as an immunosuppressive agent in Rtx patients, has been shown to down-regulate NFkβ pathway and induce apoptosis of both activated T cells and macrophages, in IL-10 knockout mice [34]. Recently, Du et al. [35] showed the importance of suppression of NFkβ by cyclosporin A and tacrolimus in renal tubular cells. In our study, out of 70 Rtx patients, 60 were receiving tacrolimus as a main immunosuppressive therapy. Despite the absence of any correlation between tacrolimus usage and sTWEAK it is plausible to suggest that decreased sTWEAK levels might be a compensatory response to the inhibitory effects of tacrolimus on the NFkB pathway (Figure 1).

Our study had two main limitations. First, this was a cross-sectional analysis of Rtx patients focusing on CIMT and sTWEAK. Second, the sample size was relatively small. This was not a prospective controlled study and therefore, we cannot draw cause-and-effect relationships from our findings.

None of our renal transplant patient died after they enrolled in this study. Hence, we could not analyze the relation between inflammation markers and mortality. However, a prospective study looking at the significance of these markers in mortality should be done.

## Conclusions

sTWEAK might have a role in the pathogenesis of ongoing atherosclerosis in Rtx patients. Rtx may not completely reverse non-traditional risk factors including inflammation and atherosclerosis. When considered together, ongoing inflammation and interactions between drugs and macrophages might explain why CKD and ESRD patients had lower sTWEAK levels than Rtx patients. Renal transplant patients should be assessed to their inflammatory and uremic status individually. There are many missing pieces in the puzzle and nothing is yet known about the clinical implications of sTWEAK in Rtx patients. Further randomized controlled trials and experimental studies investigating the exact roles of sTWEAK in the pathogenesis of atherosclerosis in renal transplantation patients are needed.

## Competing interests

The authors declare that they have no conflict of interest.

## Authors’ contributions

KT: Participated in research design and performance of the research, data analysis, participated in the writing of the paper. HZT: Participated in research design. FME: Participated in the performance of the research. AT: Participated in laboratory analysis. ZB: Participated in the performance of the research. HO: Participated in radiological measurements. AG: Participated in data analysis. EEG: Participated in the data analysis. MK: Participated in the reseach design. YS: Participated in data analysis. OO: Participated in data analysis of radiological measurements. ST: Participated in the reseach design. AC: Participated in the editing of the manuscript. All authors read and approved the final manuscript.

## Pre-publication history

The pre-publication history for this paper can be accessed here:

http://www.biomedcentral.com/1471-2369/14/144/prepub
